# Supplementary material for: Multi-layer networks reveal changes in plant-bird interactions driven by invasive species
Source: Commun Biol. 2025 Dec 2;8:1735. doi: 10.1038/s42003-025-09130-4 (PMC12672725; doi:10.1038/s42003-025-09130-4)
Supplement: Supplementary file 1 — Supplementary Information [file 42003_2025_9130_MOESM1_ESM.pdf]

SUPPLEMENTARY INFORMATION

Multi-layered networks reveal changes in plant-bird interactions driven by invasive species

Jaume Izquierdo, Dailos Hernández-Brito, Fernando Hiraldo, David García-Callejas, José L. Tella and Martina Carrete

Supplementary Methods 1. Worldwide distribution of rose-ringed and the monk parakeets

Supplementary Figure 1. Global occurrence data of the rose-ringed parakeet *Psittacula krameri* (A) and the monk parakeet *Myiopsitta monachus* (B) compiled from the eBird database[1] across their native (shaded areas[2]) and non-native (red points) ranges.

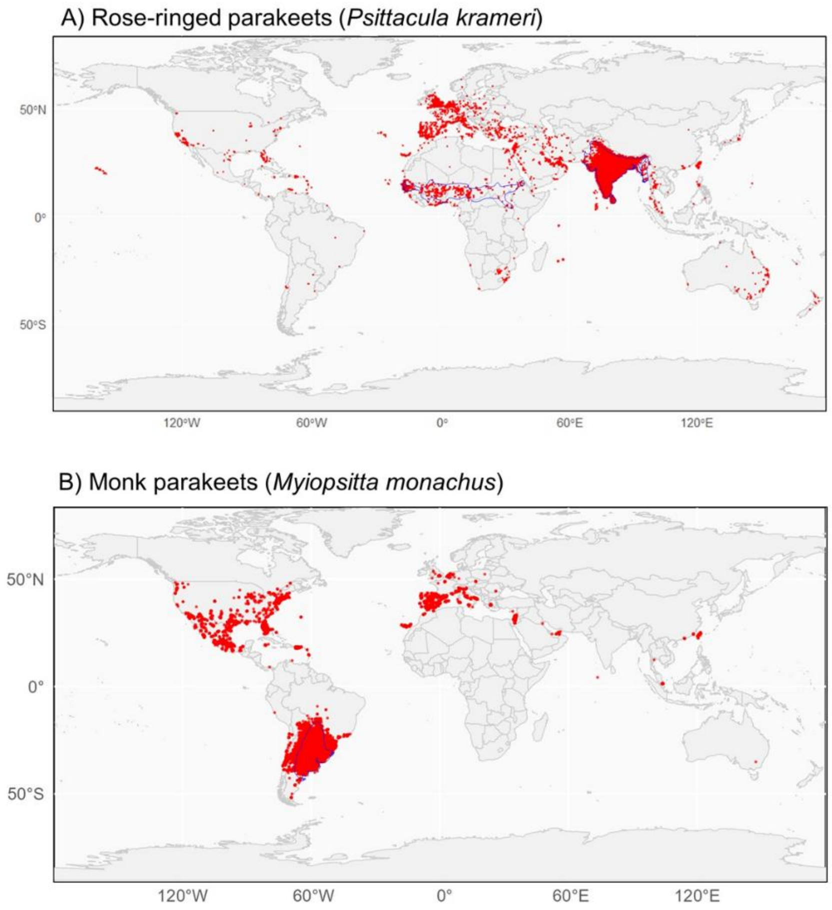

Sampling adequacy

Supplementary Figure 2. Map of the study area (outlined in red) covering the metropolitan area of Seville and surroundings (southern Spain) (indicated by a red dot in the inset map). The locations of monitored plants (i.e., where plant-bird interactions and dispersal mechanisms were recorded) are shown as blue dots.

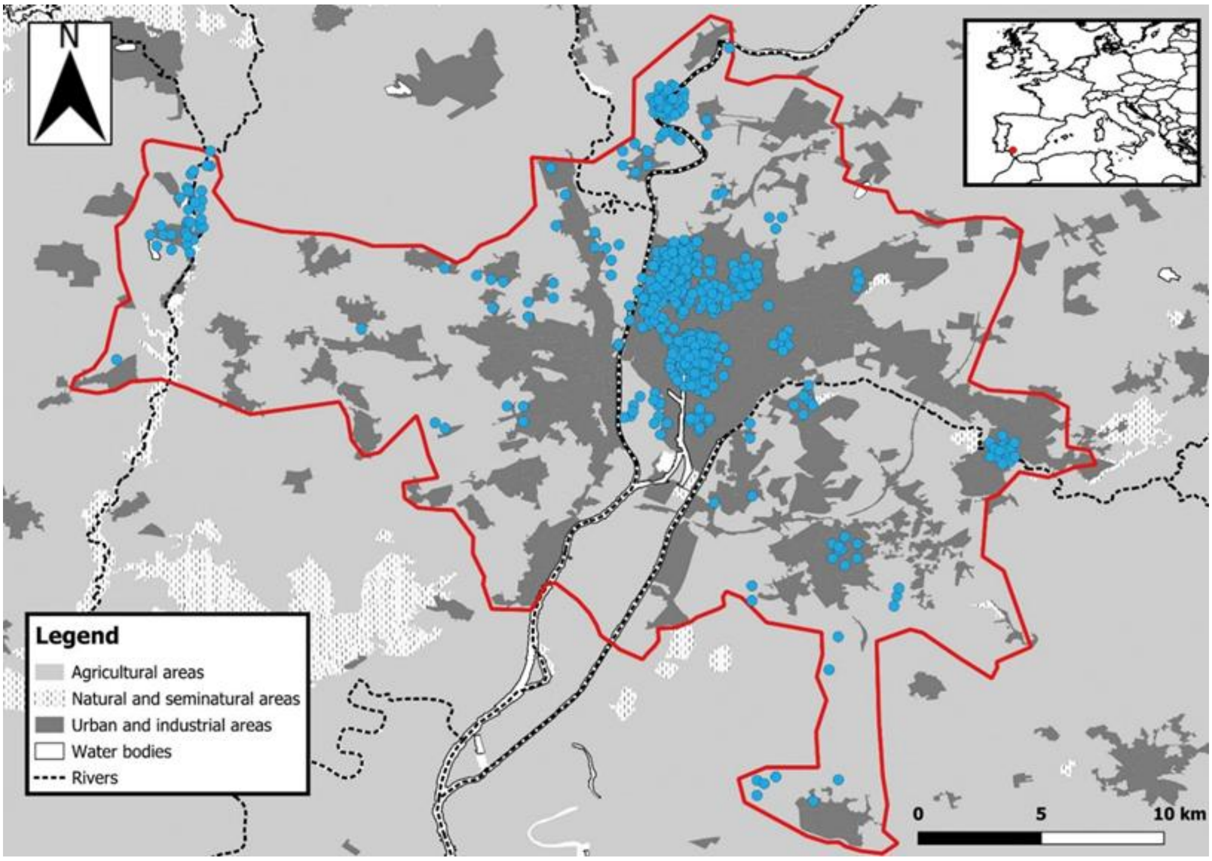

Supplementary Figure 3. Accumulation curves of the richness of birds preying and dispersing seeds of the different arboreal and shrubby plants present in the study area. Accumulation curves were calculated using the cumulative number of individuals sampled per plant species as the unit of sampling effort. Gray shading around interpolated (solid) lines represents the 95% confidence intervals. Most plants reached stabilization in their accumulation curves. The few species that did not stabilize were predominantly those utilized by parrots. However, due to their high abundance in the study area and the extensive sampling effort, we included these species in the analyses, confident they were adequately represented.

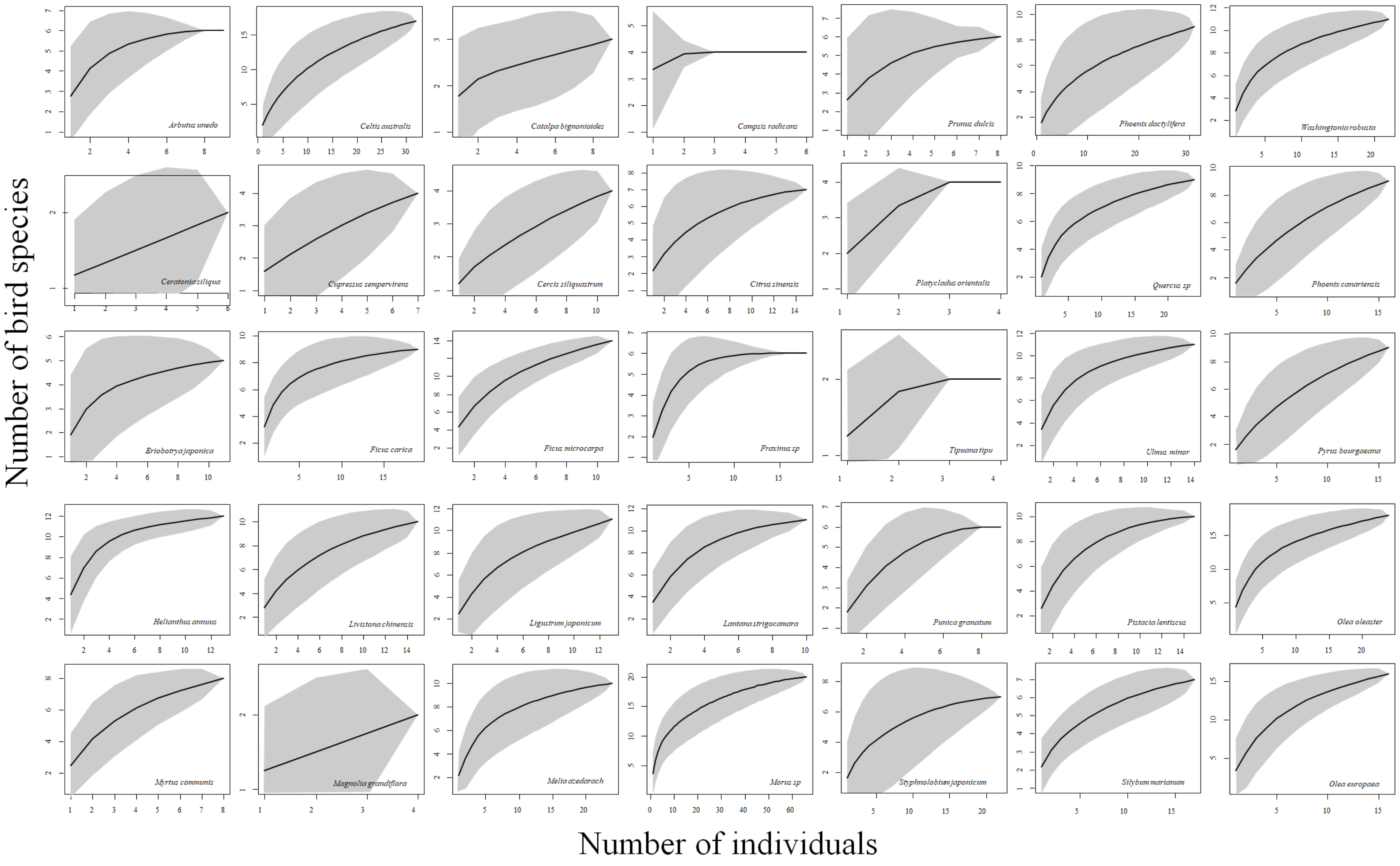
Number of bird species
Number of individuals
Arbutus unedo
Celtis australis
Catalpa bignonioides
Campsis radicans
Prunus dulcis
Phoenix dactylifera
Washingtonia robusta
Ceratonia siliqua
Cupressus sempervirens
Cercis siliquastrum
Citrus sinensis
Platycladus orientalis
Quercus sp
Phoenix canariensis
Eriobotrya japonica
Ficus carica
Ficus microcarpa
Fraxinus sp
Tipuana tipu
Ulmus minor
Pyrus bourgaeana
Helianthus annuus
Livistona chinensis
Ligustrum japonicum
Lantana strigocamara
Punica granatum
Pistacia lentiscus
Olea oleaster
Myrtus communis
Magnolia grandiflora
Melia azedarach
Morus sp
Styphnolobium japonicum
Silybum marianum
Olea europaea

Supplementary Methods 3. Classification and quantification of interspecific interactions

Interactions were classified based on the bird's actions and the fate of the seeds of each fruit (Table 1). When birds completely destroyed the seeds, the interaction was classified as seed predation. If birds consumed only the pulp of the fruit, it was considered fruit defleshing. For acorns (seeds from trees of the genus *Quercus*), partial consumption of cotyledons (less than 60%) does not damage the embryo and facilitates germination[3,4]. This is analogous to pulp removal in fleshy fruits[5,6], so these observations were classified as fruit defleshing. Interactions where birds only defleshed fruits were considered as commensalists and excluded from network analyses as it was uncertain whether this action benefits or harms germination, even though it clearly benefits the birds. Seeds that were swallowed whole were generally considered dispersed by endozoochory unless damaged while passing through the bird's digestive tract. Such instances, confirmed through faecal and regurgitate analysis, included: 1) ingestion of unripe fruits with unprotected seeds[7], 2) ingestion of ripe fruits with small seeds (< 5 mm) and soft coats (hardness 1; Supplementary Table 2) by pigeons and doves[8,9] and 3) ingestion of acorns by the wood pigeon (*Columba palumbus*), whose gizzard completely crushed them[10] (F. Hiraldo, own data). In some instances, especially with berry-like fruits, some seeds were consumed with the pulp, and the interaction was therefore classified as endozoochory. When birds carried viable seeds away from the mother plant, the interaction was classified as stomatochory if the transport was active, involving the bird carrying intact seeds in the beaks or feet, or as epizoochory if seeds were passively transported attached to the bird's body. To confirm stomatochory as an effective seed dispersal mechanism, we visually tracked the birds whenever possible and examined the fate of the seeds in situ (i.e., whether they were predated, swallowed or wasted).

To quantify the interaction strength (interaction weight, wij) of a link connecting a bird species i and a plant species j, we used the number of seeds predated or dispersed as a proportion of the total number of seeds predated, dispersed, defleshed or wasted by all bird present in a plant species, including the two parakeets. To account for the considerable variability in seed counts among fruits, we expressed bird-handled portions in fractions of fifths. For example, consuming an entire single-seeded fruit was counted as 5/5, while consuming of a part of a multi-seeded was counted as $x/5$, where x represents the portion consumed, dispersed or wasted. The inflorescences of sunflowers and thistles were treated as

fruits to quantify the seeds handled by birds. The mean number of seeds per fruit were taken from the literature (Table 2). This approach was the best approximation to assess the relative role each bird plays in the plant community, regardless of the number of total seeds consumed.

Supplementary Table 1. Plant-bird interactions used to construct the ecological networks before and after the entrance of the rose-ringed parakeet *Psittacula krameri* and the monk parakeet *Myiopsitta monachus* (pre-invasion and invasion networks, respectively). For each plant species, we included its origin (Native or Exotic to the study area), growth habit (Tree or Shrub-tree), fruit type, mean number of seeds per fruit (N seeds), number of individuals sampled for interactions (N plants) and seed harness. Fruits were classified as fleshy or dry based on the presence (or absence) of a soft, juicy mesocarp (pulp), and further divided drupaceous (i.e., single fleshy fruits with up to 10 seeds per fruit, including true drupes, pomes, hesperidia and some few-seeded berries) or berry-like (i.e., multiple fleshy fruits with more than 10 seeds per fruit, including berries, syconia and aggregates)[11]. Growth habit, geographical origin and mean number of seeds per fruit were taken from the literature (Table 2). As there is no standard measure available for all the studied plants, seed hardness was categorized into three levels representing increasing bite force required to deform or compress a seed, based on manual testing by the same person (DHB) on 20 seeds per species, including equal number of mature and immature seeds (mature/immature)[12].

**Plant species**	**Origin**	**Geographic range**	**Growth**	**Fruit type**	**N seeds/fruit**	**N plants**	**Seed hardness**	**Bird species**	**Pre-invasion network**		**Invasion network**	
									**antagonistic**	**mutualistic**	**antagonistic**	**mutualistic**
Arbutus unedo	Native	Macaronesia / Ireland / S Europe / Mediterranean	Shrub-tree	Berry-like	12.9	9	1/1	*Carduelis carduelis*	1	3	1	3
								Sylvia atricapilla	26		26	
								Curruca melanocephala	6		6	
								Fringilla coelebs	25		25	
								Passer domesticus	1	25	1	25
								Turdus merula		8		8
Campsis radicans	Exotic	E North America	Liana	Dry	696	6	1/1	*Columba livia var. dom.*			5	
								Myiopsitta monachus			6	8
								Passer domesticus			5	
								Psittacula krameri			23	21
Catalpa bignonioides	Exotic	SE USA	Tree	Dry	94.1	9	1/1	*Columba livia var. dom.*	1		1	
								Passer domesticus	8	3	8	3
								Psittacula krameri			49	3
Celtis australis	Native	S Europe / W Asia / N Africa	Tree	Drupaceous	1	32	3/2	*Columba livia var. dom.*		12		12
								Columba palumbus		7		7
								Sylvia atricapilla	1		1	
								Cyanopica cooki		3		3

Plant species	Origin	Geographic range	Growth	Fruit type	N seeds/fruit	N plants	Seed hardness	Bird species	Pre-invasion network		Invasion network	
									antagonistic	mutualistic	antagonistic	mutualistic
								Myiopsitta monachus			6	1
								Passer domesticus		1		1
								Pica pica		1		1
								Psittacula krameri			40	1
								Streptopelia decaocto		2		2
								Sturnus unicolor		3		3
								Turdus merula		3		3
Ceratonia siliqua	Native	Mediterranean	Shrub-tree	Dry	10	6	3/1	*Psittacula krameri*			52	9
								Streptopelia decaocto			1	
Cercis siliquastrum	Exotic	SE Europe / Middle East	Shrub-tree	Dry	7	11	3/1	*Columba palumbus*			1	
								Myiopsitta monachus			4	
								Psittacula krameri			47	5
								Streptopelia decaocto			1	
Citrus × aurantium	Exotic	China	Shrub-tree	Berry-like	17.82	15	2/1	*Psittacula krameri*				5
								Cyanopica cooki				1
Cupressus sempervirens	Exotic	Aegean	Tree	Dry	152.1	7	2/2	*Chloris chloris*			6	
								Myiopsitta monachus			6	5
								Psittacula krameri			22	21
								Streptopelia decaocto			2	
Eriobotrya japonica	Exotic	W China / Japan	Shrub-tree	Drupaceous	1.5	11	1/1	*Myiopsitta monachus*				1
								Psittacula krameri				5
Ficus carica	Native	Mediterranean / Middle East / Pakistan	Tree	Berry-like	1215	19	1/1	*Sylvia atricapilla*	1		1	
								Myiopsitta monachus				28
								Oriolus oriolus	1		1	
								Passer domesticus		8		8
								Passer hispaniolensis		1		1
								Psittacula krameri				44
								Sturnus unicolor		4		4
								Sylvia borin		1		1
								Turdus merula		2		2
Ficus microcarpa	Exotic	Indian subcontinent / SE Asia	Tree	Berry-like	168	11	1/1	*Coloeus monedula*	5		5	
								Columba livia var. dom.	2		2	
								Columba palumbus	6		6	
								Sylvia atricapilla	13		13	
								Curruca melanocephala	1		1	

Plant species	Origin	Geographic range	Growth	Fruit type	N seeds/fruit	N plants	Seed hardness	Bird species	Pre-invasion network		Invasion network	
									antagonistic	mutualistic	antagonistic	mutualistic
								Myiopsitta monachus				6
								Passer domesticus	1	2	1	2
								Passer hispaniolensis		1		1
								Pica pica		1		1
								Psittacula krameri				27
								Streptopelia decaocto	1		1	
								Sturnus unicolor		6		6
								Sylvia borin		1		1
								Turdus merula		23		23
Fraxinus sp	Native	*F. angustifolia*: Europe /SW Asia / NW África	Tree	Dry	1	18	1/1	*Chloris chloris*	8		8	
								Fringilla coelebs	10	1	10	1
								Myiopsitta monachus			49	
		F. excelsior: S Europe / W Asia						*Pica pica*	1	1	1	1
								Psittacula krameri			23	
								Streptopelia decaocto	2		2	
Helianthus annuus	Exotic	NW North America	Herb	Dry	591.6	13	1/1	*Carduelis carduelis*	5		5	
								Chloris chloris	6		6	
								Columba livia var. dom.	8		8	
								Columba palumbus	3		3	
								Cyanopica cooki	2		2	
								Linnaria cannabina	2		2	
								Passer domesticus	3		3	
								Passer hispaniolensis	5		5	
								Pica pica	2		2	
								Psittacula krameri			43	
								Serinus serinus	2		2	
								Streptopelia decaocto	19		19	
Lantana strigocamara	Exotic	Caribbean / Central / America / N South America	Shrub	Drupaceous	1	10	2/1	*Chloris chloris*	26		26	
								Coloeus monedula	4		4	
								Columba livia var. dom.		12		12
								Sylvia atricapilla	12		12	
								Curruca melanocephala	6		6	
								Passer domesticus	1		1	
								Streptopelia decaocto		6		6
								Sturnus unicolor		2		2

Plant species	Origin	Geographic range	Growth	Fruit type	N seeds/fruit	N plants	Seed hardness	Bird species	Pre-invasion network		Invasion network	
									antagonistic	mutualistic	antagonistic	mutualistic
								Sylvia borin		1		1
								Turdus merula		15		15
Ligustrum japonicum	Exotic	Korea / Japan	Shrub-tree	Drupaceous	1	13	1/1	*Carduelis carduelis*	1		1	
								Chloris chloris	22		22	
								Columba livia var. dom.	1		1	
								Sylvia atricapilla	1		1	
								Curruca melanocephala	1		1	
								Myiopsitta monachus			32	2
								Passer domesticus	1		1	
								Psittacula krameri			30	5
								Sturnus unicolor		1		1
								Turdus merula		1		1
								Turdus philomelos	1		1	
Livistona chinensis	Exotic	SE China / Japan	Tree	Drupaceous	1	15	3/2	*Coloeus monedula*	1		1	
								Myiopsitta monachus				1
								Passer domesticus		1		1
								Psittacula krameri				18
								Turdus merula		13		13
Melia azedarach	Exotic	Indian subcontinent / SE Asia / Japan / N Australia	Tree	Drupaceous	3.61	24	3/2	*Columba palumbus*		5		5
								Sylvia atricapilla	2		2	
								Cyanopica cooki		16		16
								Myiopsitta monachus			18	
								Psittacula krameri			27	1
								Sturnus unicolor		1		1
								Turdus merula		5		5
								Turdus philomelos	5		5	
Morus sp	Exotic	*M. alba*: CE China / Korea<br>*M. nigra*: Anatolia	Tree	Berry-like	17.9	66	1/1	*Chloris chloris*	4	1	4	1
								Coloeus monedula	1		1	
								Columba livia var. dom.	1		1	
								Columba palumbus	1		1	
								Sylvia atricapilla	1		1	
								Curruca melanocephala	1		1	
								Cyanopica cooki	1	8	1	8
								Linnaria cannabina	1	1	1	1
								Myiopsitta monachus			7	7

Plant species	Origin	Geographic range	Growth	Fruit type	N seeds/fruit	N plants	Seed hardness	Bird species	Pre-invasion network		Invasion network	
									antagonistic	mutualistic	antagonistic	mutualistic
								Oriolus oriolus	1		1	
								Passer domesticus	1	1	1	1
								Passer hispaniolensis		1		1
								Passer montanus		1		1
								Pica pica		1		1
								Psittacula krameri			3	10
								Serinus serinus	1	1	1	1
								Streptopelia decaocto	1		1	
								Sturnus unicolor	1	16	1	16
								Sylvia borin	1	8	1	8
								Turdus merula	1	9	1	9
Myrtus communis	Native	Mediterranean	Shrub-tree	Berry-like	5.2	8	1/1	*Chloris chloris*	10	10	10	10
								Columba livia var. dom.	3		3	
								Sylvia atricapilla	3		3	
								Curruca melanocephala	1		1	
								Fringilla coelebs	1		1	
								Passer domesticus	1	3	1	3
								Psittacula krameri			9	41
								Turdus merula		10		10
Olea europaea	Native	Mediterranean	Shrub-tree	Drupaceous	1	16	3/2	*Coloeus monedula*	1		1	
								Cyanopica cooki		1		1
								Myiopsitta monachus				1
								Passer domesticus		1		1
								Pica pica		1		1
								Psittacula krameri				15
								Sturnus unicolor		1		1
								Turdus merula		4		4
Olea europaea europaea	Native	Mediterranean	Shrub-tree	Drupaceous	1	24	3/2	*Chloris chloris*		1		1
								Columba livia var. dom.		1		1
								Sylvia atricapilla	5		5	
								Curruca melanocephala	1		1	
								Cyanopica cooki		4		4
								Fringilla coelebs		1		1
								Myiopsitta monachus				1
								Pica pica		2		2

Plant species	Origin	Geographic range	Growth	Fruit type	N seeds/fruit	N plants	Seed hardness	Bird species	Pre-invasion network		Invasion network	
									antagonistic	mutualistic	antagonistic	mutualistic
								Psittacula krameri			15	2
								Sturnus unicolor		3		3
								Turdus merula		9		9
								Turdus philomelos	9		9	
Phoenix canariensis	Exotic	Canary Islands	Tree	Drupaceous	1	43	3/2	*Myiopsitta monachus*			5	1
								Passer domesticus		1		1
								Psittacula krameri			20	10
								Sturnus unicolor		2		2
								Turdus merula		7		7
Phoenix dactylifera	Exotic	NE Sahara / Middle East	Tree	Drupaceous	1	31	3/2	*Cyanopica cooki*		2		2
								Myiopsitta monachus			4	1
								Psittacula krameri			57	3
Pistacia lentiscus	Native	Canary Islands / Mediterranean	Shrub-tree	Drupaceous	1	15	2/1	*Carduelis carduelis*	2		2	
								Chloris chloris	18	1	18	1
								Sylvia atricapilla	21		21	
								Curruca communis	2		2	
								Curruca melanocephala	8		8	
								Cyanopica cooki		26		26
								Passer domesticus	2	1	2	1
								Sylvia borin		9		9
								Turdus merula		9		9
								Turdus philomelos	1		1	
Platycladus orientalis	Exotic	China	Shrub-tree	Dry	251.5	4	2/1	*Chloris chloris*			10	
								Myiopsitta monachus			55	
								Passer domesticus			3	
								Psittacula krameri			17	2
Prunus dulcis	Exotic	Balkans / SW Asia / N Africa	Tree	Dry	1	8	3/3	*Myiopsitta monachus*			23	
								Psittacula krameri			45	11
Punica granatum	Exotic	Irano-Turanian	Tree	Berry-like	384.1	9	2/1	*Sylvia atricapilla*	17		17	
								Curruca melanocephala	10		10	
								Myiopsitta monachus			25	4
								Passer domesticus	1	10	1	10
								Psittacula krameri			4	3
								Turdus merula		7		7
Pyrus bourgaeana	Native	Iberian Peninsula / NW Africa	Tree	Drupaceous	7.9	16	1/1	*Cyanopica cooki*		2		2

Plant species	Origin	Geographic range	Growth	Fruit type	N seeds/fruit	N plants	Seed hardness	Bird species	Pre-invasion network		Invasion network	
									antagonistic	mutualistic	antagonistic	mutualistic
								Myiopsitta monachus				2
								Psittacula krameri			17	6
								Streptopelia decaocto			1	
Quercus sp	Native	*Q. ilex*: Mediterranean	Tree	Dry	1	24	1/1	*Coloeus monedula*	3		3	
		Q. suber: W Mediterranean						*Columba palumbus*	6		6	
								Myiopsitta monachus			7	5
								Pica pica		13		13
								Psittacula krameri			9	17
								Turdus merula		1		1
Silybum marianum	Native	Mediterranean / SW Asia / Indian	Herb	Dry	150	17	2/1	*Carduelis carduelis*	11		11	
		subcontinent						*Chloris chloris*	24		24	
								Linnaria cannabina	1		1	
								Passer domesticus	1		1	
								Passer hispaniolensis	3		3	
								Psittacula krameri			6	46
								Serinus serinus	1		1	
Styphnolobium japonicum	Exotic	E China	Tree	Drupaceous	3.5	22	2/1	*Columba livia var. dom.*	1		1	
								Myiopsitta monachus			42	1
								Psittacula krameri			43	3
								Streptopelia decaocto	1		1	
								Sturnus unicolor		1		1
								Turdus merula		1		1
Tipuana tipu	Exotic	Bolivia / N Southern Cone	Tree	Dry	1.4	4	3/2	*Myiopsitta monachus*			66	
								Psittacula krameri			17	2
Ulmus minor	Native	Europe / NW Asia	Tree	Dry	1	14	1/1	*Carduelis carduelis*	10		10	
								Chloris chloris	21		21	
								Columba palumbus	3		3	
								Linnaria cannabina	6		6	
								Myiopsitta monachus			7	
								Passer domesticus	3		3	
								Passer hispaniolensis	1		1	
								Passer montanus	1		1	
								Psittacula krameri			21	2
								Serinus serinus	12		12	
								Streptopelia decaocto	7		7	

Plant species	Origin	Geographic range	Growth	Fruit type	N seeds/fruit	N plants	Seed hardness	Bird species	Pre-invasion network		Invasion network	
									antagonistic	mutualistic	antagonistic	mutualistic
Washingtonia robusta	Exotic	SW USA / N Mexico	Tree	Drupaceous	1	22	3/2	*Coloeus monedula*	1		1	
								Columba livia var. dom.		3		3
								Sylvia atricapilla	7		7	
								Curruca melanocephala	1		1	
								Cyanopica cooki		6		6
								Myiopsitta monachus				1
								Passer domesticus		2		2
								Pica pica		2		2
								Psittacula krameri				4
								Sturnus unicolor		10		10
								Turdus merula		24		24

Table 2. List of bibliographic references used to obtain plant characteristics and origin.

Alcolea M, Durigan, G, Christianini, AV. 2022. Prescribed fire enhances seed removal by ants in a Neotropical savanna. Biotropica, 54, 125-134.
Al-Snafi AE. 2016. Medical importance of Cupressus sempervirens-A review. IOSR Journal of Pharmacy, 6, 66-76.
Amimi N, Ghouil H, Zitouna-Chebbi R, et al. 2023. Intraspecific variation of Quercus ilex L. seed morphophysiological traits in Tunisia reveals a trade-off between seed germination and shoot emergence rates along a thermal gradient. Annals of Forest Science, 80, 12.
Anand J, Rawat JS, Rawat V, et al. 2022. Climatic and Altitudinal Variation in Physicochemical Properties of Citrus sinensis in India. Land, 11, 2033.
Aronne G, Wilcock CC. 1994. First evidence of myrmecochory in fleshy-fruited shrubs of the Mediterranean region. New Phytologist, 127, 781-788.
Aydın E, Bostan SZ, Şen SM, et al. 2015, September. Selection of mulberry (Morus alba) in Artvin. In Third Balkan Symposium on Fruit Growing. pp. 16-18.
Bain A, Harrison RD, Schatz B. 2014. How to be an ant on figs. Acta Oecologica, 57, 97-108.
Balaguer-Romano R, Barea-Marquez A, Ocaña-Calahorro FJ, et al. 2021. The potential role of synzoochory in the naturalization of almond tree. Basic and Applied Ecology, 50, 97-106.
Barbosa KC, Pizo MA. 2006. Seed rain and seed limitation in a planted gallery forest in Brazil. Restoration Ecology, 14, 504-515.

Batlle, I. and J. Tous. 1997. Carob tree. Ceratonia siliqua L. Promoting the conservation and use of underutilized and neglected crops. 17. Institute of Plant Genetics and Crop Research, Gatersleben/International Plant Genetic Resources Institute, Rome, Italy. Plant
Castroviejo S. 1986-2012. Flora iberica 1-8, 10-15, 17-18, 21. Real Jardín Botánico, CSIC, Madrid.
Chachalis D, Reddy KN. 2000. Factors affecting Campsis radicans seed germination and seedling emergence. Weed science, 48, 212-216.
Chen L, Deng X, Ding M, et al. 2014. Geographic variation in traits of fruit stones and seeds of Melia azedarach. Journal of Beijing Forestry University, 36, 15-20.
Ciccarelli D, Andreucci AC, Pagni AM, Garbari F. 2005. Structure and development of the elaiosome in Myrtus communis L.(Myrtaceae) seeds. Flora-Morphology, Distribution, Functional Ecology of Plants, 200, 326-331.
Colic S, Rakonjac V, Zec G, et al. 2012. Morphological and biochemical evaluation of selected almond [Prunus dulcis (Mill.) DA Webb] genotypes in northern Serbia. Turkish Journal of Agriculture and Forestry, 36, 429-438.
Coşkuner Y, Gökbudak A. 2016. Dimensional specific physical properties of fan palm fruits, seeds and seed coats (Washingtonia robusta). doi: 10.1515/intag-2016-0004
Dickson J G. 1990. Cercis canadensis L. Eastern redbud. Silvics of North America, 2, 266-269. https://www.srs.fs.usda.gov/pubs/misc/ag_654/vol-ume_2/cercis/canadensis.htm
El-haak, MA, Atta BM, Abd Rabo FF. 2015. Seed yield and important seed constituents for naturally and cultivated milk thistle (Silybum marianum) plants†. The Egyptian Journal of Experimental Biology (Botany), 11, 141-146.
Etebu E, Nwauzoma AB. 2014. A review on sweet orange (Citrus sinensis L Osbeck): health, diseases and management. American Journal of Research Communication, 2, 33-70.
Fedriani JM, Delibes M. 2009. Functional diversity in fruit-frugivore interactions: a field experiment with Mediterranean mammals. Ecography, 32, 983-992.
Göksoy AT, Türkeç A, Turan ZM. 1999. A Research on the Analysis of Heterotic Effects for Certain Agronomical Characters in Cross Population of Sunflower (Helianthus annuus L. Turkish Journal of Agriculture and Forestry, 23, 247-256.
Gressler E, Pizo MA, Morellato LPC. 2006. Polinização e dispersão de sementes em Myrtaceae do Brasil. Brazilian Journal of Botany, 29, 509-530.

Gunes MEH, Cekic C. 2004. Some chemical and physical properties of fruits of different mulberry species commonly grown in Anatolia, Turkey. Asian J. Chem, 16, 1849-1855.
Hashemi S, Khadivi A. 2020. Morphological and pomological characteristics of white mulberry (Morus alba L.) accessions. Scientia Horticulturae, 259, 108827.
Heiser CB. 1976. The sunflower. University of Oklahoma Press, Norman. https://id.oclc.org/worldcat/entity/E39PBJyxcWXTrvbYJCD7gfyGHC/
Hosseini AS, Akramian M, Khadivi A, Salehi-Arjmand H. 2018. Phenotypic and chemical variation of black mulberry (Morus nigra) genotypes. Industrial Crops and Products, 117, 260-271.
Hussain A. 2009. Study of seasonal biomass productivity and nutritional quality of major forage species in subtropical sub humid rangelands of district Chakwal. PhD Thesis, Pir Mehr Ali Shah Arid Agriculture University, Rawalpindi, 379 p.
Jordano P. 1995. Angiosperm fleshy fruits and seed dispersers: a comparative analysis of adaptation and constraints in plant-animal interactions. The American Naturalist, 145, 163-191.
Kaufmann S, McKey DB, Hossaert-McKey M, et al. 1991. Adaptations for a two-phase seed dispersal system involving vertebrates and ants in a hemiepiphytic fig (Ficus microcarpa: Moraceae. American Journal of Botany, 78, 971-977.
Kaveh M, Tavassoli A, Azadi R, Memariani F. 2014. Morphology and micromorphology of the genus Fraxinus L. in Iran. The Iranian Journal of Botany, 20, 188-200.
Khadivi A, Arab M. 2021. Identification of the superior genotypes of pomegranate (Punica granatum L.) using morphological and fruit characters. Food Science & Nutrition, 9, 4578-4588.
Kılıçkan A, Güner METİN. 2008. Physical properties and mechanical behavior of olive fruits (Olea europaea L.) under compression loading. Journal of Food Engineering, 87, 222-228.
Liao T, Liu G, Guo L, et al. 2021. Bud Initiation, microsporogenesis, megasporogenesis, and cone development in Platycladus orientalis. HortScience, 56, 85-93.
Lim TK. 2013. Styphnolobium japonicum. In Edible Medicinal and Non-Medicinal Plants: Volume 7, Flowers. pp. 906-924. Dordrecht: Springer Netherlands.

Liu Y, Ma Q, Liu H, et al. 2014. Phenotypic diversity of fruits and seeds of Magnolia grandiflora superior trees. Acta Agriculturae Shanghai, 30, 65-67.
Lososová Z, Axmanová I, Chytrý M, et al. 2023. Seed dispersal distance classes and dispersal modes for the European flora. Global Ecology and Biogeography, 32, 1485-1494.
Lovisetto A, Masiero S, Rahim MA, et al. 2015. Fleshy seeds form in the basal angiosperm Magnolia grandiflora and several MADS-box genes are expressed as fleshy seed tissues develop. Evolution, Development, 17, 82-91.
Mathew G, Skaria BP, Joseph A. 2011. Standardization of conventional propagation techniques for four medicinal species of genus Ficus Linn.
Monge RA, Martínez MF. 2012. Myrtus communis L. https://www.researchgate.net/profile/Reyes_Alejano/publication/257941420_My rtus_communis_L/links/02e7e526659bc3116b000000.pdf
Mulas M. 1999. Characterisation of olive wild ecotypes. In III International Symposium on Olive Growing. Acta Horticulturae, 474, 121–124.
Olsen RT, Kirkbride Jr JH. 2017. Taxonomic revision of the genus Catalpa (Bignoniaceae. Brittonia, 69, 387-421.
Olsen RT. 2007. Utilizing Polyploidy for Developing Improved Nursery Crops: Restoring Fertility in Wide Hybrids, Limiting Fertility of Invasive Species, Embryo Culture of Triploids, Pest Resistance, and Inheritance of Ornamental Traits. http://www.lib.ncsu.edu/resolver/1840.16/3495
Patterson KJ. 1990. Effects of pollination on fruit set, size, and quality in feijoa (Acca sellowiana (Berg) Burret. New Zealand Journal of Crop and Horticultural Science, 18, 127-131.
Pece MG, de Benítez CG, Acosta M, et al. 2010. Germinación de Tipuana tipu (Benth.) O. Kuntze (tipa blanca) en condiciones de laboratorio. Quebracho-Revista de Ciencias Forestales, 18, 5-15. http://www.redalyc.org/articulo.oa?id=48118695001
POWO. 2024. Plants of the World Online. Facilitated by the Royal Botanic Gardens, Kew. Published on the Internet; https://powo.science.kew.org/
Ramaswami G, Somnath P, Quader S. 2017. Plant-disperser mutualisms in a semi-arid habitat invaded by Lantana camara L. Plant Ecology, 218, 935-946.
Ramírez-Valiente JA, Valladares F, Gil L, Aranda I. 2009. Population differences in juvenile survival under increasing drought are mediated by seed size in cork oak (Quercus suber L.. Forest Ecology and Management, 257, 1676-1683.
Sebastián-González E, Hiraldo F, Blanco G, et al. 2019. The extent, frequency and ecological functions of food wasting by parrots. Scientific Reports, 9, 15280.

Sękiewicz K, Boratyńska K, Dagher-Kharrat MB, et al. 2016. Taxonomic differentiation of Cupressus sempervirens and C. atlantica based on morphometric evidence. Systematics and Biodiversity, 14, 494-508.
Sidina MM, El Hansali M, Wahid N, et al. 2009. Fruit and seed diversity of domesticated carob (Ceratonia siliqua L.) in Morocco. Scientia horticulturae, 123, 110-116.
Simozrag A, Chala A, Djerouni A, Bentchikou ME. 2016. Phenotypic diversity of date palm cultivars (Phoenix dactylifera L.) from Algeria. Gayana Botanica, 73, 42-53.
Spennemann DH, Pike M, Robinson W. 2020. Germination rates of old and fresh seeds and their implications on invasiveness of the ornamental Canary Islands date palm (Phoenix canariensis. European Journal of Ecology, 6.
Spennemann DH. 2020. Palms fanning out: a review of the ecological provisioning services provided by Washingtonia filifera and W. robusta in their native and exotic settings. Plant Ecology, Diversity, 13, 289-324.
Starr F, Starr K, Loope L. 2003. Ficus microcarpa. Chinese Banyan, Moraceae. United States Geological Survey-Biological Resources Division Haleakala Field Station, Maui, Hawai'i.
Xolmurotov M. 2023. Fruit and seed morphology of Japanese ligustrum (Ligustrum japonicum) and Chinese ligustrum (Ligustrum sinense. Journal of Agriculture, Horticulture, 3, 61-64.
Yücesan Z, Ak N. 2021. Effects of seed morphology and growing media on germination percentage and growth of field elm (Ulmus minor Miller subsp Ulmus minor Miller subsp Ulmus minorminor. Austrian Journal of Forest Science, 138, 1.

Supplementary Table 3. Geographic range[13] of bird species recorded feeding on fruits and seeds during the study period. Non-native bird species in the study area are indicated with an asterisk.

Order	Family	Bird species	Geographic range
**Columbiformes**	Columbidae	*Columba livia* var. *dom.*	Macaronesia-Eurasia-N Africa-Sahel
		Columba palumbus	Europe / Near East
		Streptopelia decaocto	Macaronesia / Eurasia / NW Africa
**Passeriformes**	Corvidae	*Coloeus monedula*	Europe / NW Asia
		Cyanopica cooki	Iberian Peninsula
		Pica pica	Eurasia / NW Africa
	Fringillidae	*Carduelis carduelis*	Macaronesia / Europe / W Asia / Irano-Turanian
		Chloris chloris	Macaronesia / Europe / NW Asia
		Fringilla coelebs	Europe / NW Asia
		Linaria cannabina	Macaronesia / Europe / N Africa / NW Asia / Irano-Turanian
		Serinus serinus	Europe / Mediterranean
	Oriolidae	*Oriolus oriolus*	Europe / NW Asia / C-SE Africa
	Passeridae	*Passer domesticus*	Europe / N Africa / NW Asia / Indian subcontinent
		Passer hispaniolensis	Macaronesia / Mediterranean / Middle East / NW India
		Passer montanus	Europe / W-SE Asia

	Sturnidae	*Sturnus unicolor*	W Mediterranean
	Sylviidae	*Curruca communis*	Europe / NW Asia / S Sahel / CW Africa
		Curruca melanocephala	Macaronesia / Mediterranean / Sahel
		Sylvia atricapilla	Macaronesia / Europe / N Africa / NW Asia / Ecuatorial Africa
		Sylvia borin	Europa / NW Asia / Sub-Saharan Africa
	Turdidae	*Turdus merula*	Europe / N Africa / Middle East / W China
		Turdus philomelos	Europa / N Africa / NW Asia
**Psittaciformes**	Psittacidae	*Myiopsitta monachus*	S Bolivia / N Southern Cone
	Psittaculidae	*Psittacula krameri*	Indian subcontinent / S Sahel

Supplementary Table 4. Food facilitation events recorded during the study period. Facilitation is defined as any interaction in which a species -primarily parakeets, Iberian magpies *Cyanopica cooki* or humans- enhances fruit or seed access for other bird species by discarding, partially consuming or modifying food items, thereby making them more available, accessible, or easier to consume.

**Seed predation**	**N fruits**	**Facilitating spp**
Chloris chloris - Cupressus sempervirens	41	Parakeets
Chloris chloris - Platycladus orientalis	27	Parakeets
Columba livia var. dom. - Campsis radicans	40	Parakeets

Columba palumbus - Cercis siliquastrum	2	Parakeets
Passer domesticus - Arbutus unedo	1	Unknown
Passer domesticus - Campsis radicans	52	Parakeets
Passer domesticus - Ficus microcarpa	8	Human
Passer domesticus - Platycladus orientalis	14	Parakeets
Streptopelia decaocto - Ceratonia siliqua	1	Parakeets
Streptopelia decaocto - Cercis siliquastrum	36	Parakeets
Streptopelia decaocto - Cupressus sempervirens	8	Parakeets
**Fruit defleshing**	**N fruits**	**Facilitator spp**
Columba livia var. dom. - Quercus sp	32	Parakeets
Passer montanus - Quercus sp	1	Parakeets
Streptopelia decaocto - Quercus sp	53	Parakeets
Cyanopica cooki - Citrus sinensis	13	Parakeets
Passer domesticus - Citrus sinensis	84	Parakeets
Passer hispaniolensis - Citrus sinensis	2	Parakeets
Sylvia atricapilla - Citrus sinensis	38	Parakeets
Sylvia atricapilla - Pyrus bourgaeana	11	*Cyanopica cooki*
Sylvia borin - Pyrus bourgaeana	2	*Cyanopica cooki*
Curruca melanocephala - Citrus sinensis	8	Parakeets
Turdus merula - Citrus sinensis	3	Parakeets
Turdus merula - Phoenix dactylifera	4	*Cyanopica cooki*

Turdus philomelos - Phoenix dactylifera	4	*Cyanopica cooki*
**Seed dispersal**	**N fruits**	**Facilitator spp**
Cyanopica cooki - Citrus sinensis	5	Parakeets

Supplementary Methods 4. Species roles

Supplementary Figure 4. Contribution to nestedness by birds (left panel) and plants (right panel) in the invasion multilayer network. Species with positive values make a greater contribution to nestedness than those with negative values.

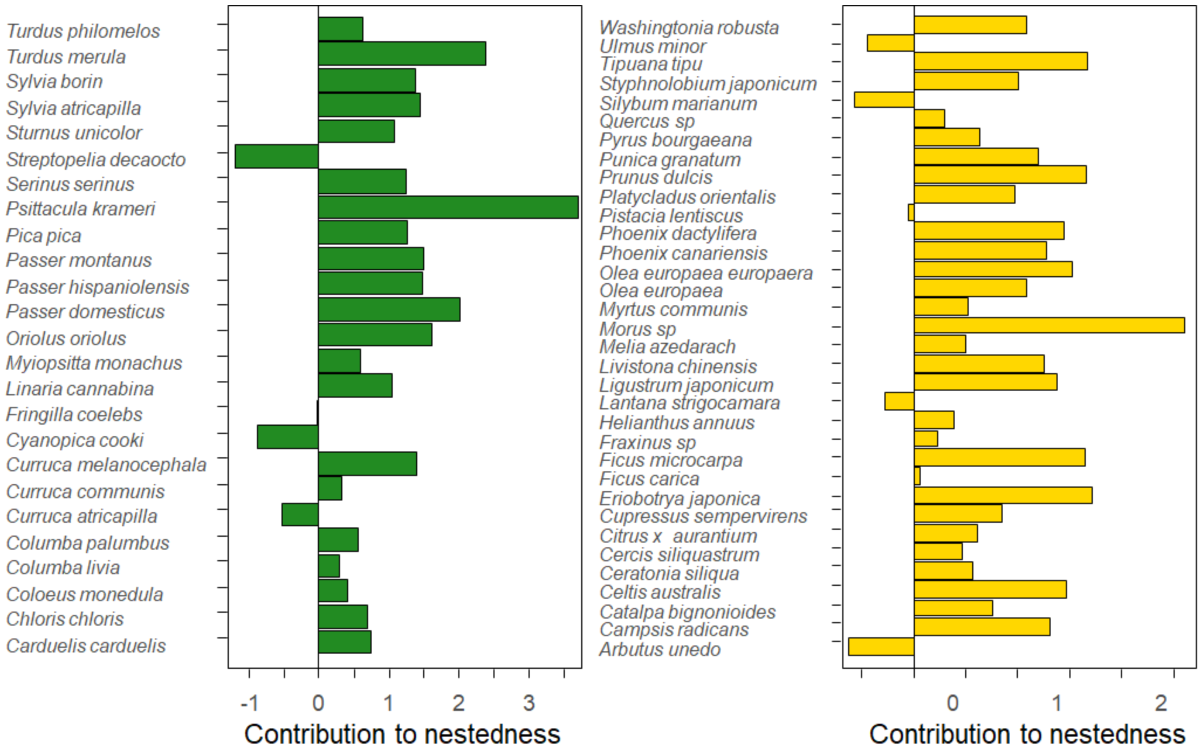

Supplementary References

1 Sullivan, B.L., Wood, M.J., Iliff, R.E. Bonney, D. Fink & Kelling, S. (2009) eBird: a citizen-based bird observation network in the biological sciences. Biological Conservation 142: 2282-2292.

2 BirdLife International (2018) Bird species distribution maps of the world. Version 7.0. Available at https://datazone.birdlife.org/species/requestdis

3 Mancilla-Leytón, J.M., Cambrollé, J. & Vicente, Á.M. (2012) The impact of the common rabbit on cork oak regeneration in SW Spain. Plant Ecology, 213, 1503-1510.

4 Leiva, M.J., Pérez-Romero, J.A. & Mateos-Naranjo, E. (2018) The effect of simulated damage by weevils on Quercus ilex subsp. Ballota acorns germination, seedling growth and tolerance to experimentally induced drought. Forest Ecology and Management 409, 740-748

5 Fedriani, J.M. & Delibes, M. (2013) Pulp feeders alter plant interactions with subsequent animal associates. Journal of Ecology, 101, 1581-1588.

6 Baskin, J.M. & Baskin, C.C. (2014) What kind of seed dormancy might palms have? Seed Science Research, 24, 17-22.

7 Blanco, G. et al. (2016) Internal seed dispersal by parrots: an overview of a neglected mutualism. *PeerJ* **4**, e1688.

8 Lambert, F.R. (1989) Pigeons as seed predators and dispersers of figs in a Malaysian lowland forest. Ibis, 131, 521-527.

9 Marrero, P. & Nogales, M. (2021) Trophic strategies of two sympatric endemic pigeons in insular ecosystems: a framework for understanding spatiotemporal frugivory interactions. Journal of Avian Biology, 52(10).

10 Ouden, J.D., Jansen, P.A. & Smit, R. (2005) Jays, mice and oaks: predation and dispersal of Quercus robur and Q. petraea in North-western Europe. In (M. Forget, J.E Lambert, P.E. Hulme, S.B. Vander Wall, eds) Seed fate: predation, dispersal and seedling establishment. CABI, Oxforshire.

11 Guimarães Jr, P.R., Galetti, M. & Jordano, P. (2008) Seed dispersal anachronisms: rethinking the fruits extinct megafauna ate. PloS one, e1745.

12 Alcaraz-Mármol, F., Calín-Sánchez, Á., Nuncio-Jáuregui, N., Carbonell-Barrachina, Á. A., Hernández, F., & Martínez, J. J. (2015). Classification of pomegranate cultivars according to their seed hardness and wood perception. Journal of Texture Studies, 46(6), 467-474.

13 Billerman, S. M., B. K. Keeney, G. M. Kirwan, F. Medrano, N. D. Sly & M. G. Smith, Editors (2025) Birds of the World. Cornell Laboratory of Ornithology, Ithaca, NY, USA. https://birdsoftheworld.org/bow/home
