# Supplementary material for: Multi-layer networks reveal changes in plant-bird interactions driven by invasive species
Source: Commun Biol. 2025 Dec 2;8:1735. doi: 10.1038/s42003-025-09130-4 (PMC12672725; doi:10.1038/s42003-025-09130-4)
Supplement: Supplementary file 2 — Description of Additional Supplementary Files [file 42003_2025_9130_MOESM2_ESM.pdf]

Description of Additional Supplementary Files

File: Supplementary Data

Description: Source data for figures in the study.
