# Supplementary material for: Multi-layer networks reveal changes in plant-bird interactions driven by invasive species
Source: Commun Biol. 2025 Dec 2;8:1735. doi: 10.1038/s42003-025-09130-4 (PMC12672725; doi:10.1038/s42003-025-09130-4)
Supplement: Supplementary file 4 — Reporting Summary [file 42003_2025_9130_MOESM4_ESM.pdf]

nature portfolio

Corresponding author(s): Dailos Hernández Brito

Last updated by author(s): Oct 15, 2025

Reporting Summary

Nature Portfolio wishes to improve the reproducibility of the work that we publish. This form provides structure for consistency and transparency in reporting. For further information on Nature Portfolio policies, see our Editorial Policies and the Editorial Policy Checklist.

Statistics

For all statistical analyses, confirm that the following items are present in the figure legend, table legend, main text, or Methods section.

n/a	Confirmed	
☐	☒	The exact sample size (*n*) for each experimental group/condition, given as a discrete number and unit of measurement
☐	☒	A statement on whether measurements were taken from distinct samples or whether the same sample was measured repeatedly
☐	☒	The statistical test(s) used AND whether they are one- or two-sided<br>*Only common tests should be described solely by name; describe more complex techniques in the Methods section.*
☐	☒	A description of all covariates tested
☐	☒	A description of any assumptions or corrections, such as tests of normality and adjustment for multiple comparisons
☐	☒	A full description of the statistical parameters including central tendency (e.g. means) or other basic estimates (e.g. regression coefficient) AND variation (e.g. standard deviation) or associated estimates of uncertainty (e.g. confidence intervals)
☐	☒	For null hypothesis testing, the test statistic (e.g. *F*, *t*, *r*) with confidence intervals, effect sizes, degrees of freedom and *P* value noted<br>*Give P values as exact values whenever suitable.*
☒	☐	For Bayesian analysis, information on the choice of priors and Markov chain Monte Carlo settings
☒	☐	For hierarchical and complex designs, identification of the appropriate level for tests and full reporting of outcomes
☒	☐	Estimates of effect sizes (e.g. Cohen's *d*, Pearson's *r*), indicating how they were calculated

Our web collection on statistics for biologists contains articles on many of the points above.

Software and code

Policy information about availability of computer code

Data collection: No software was used

Data analysis: Core Team. R: A language and environment for statistical computing. R Foundation for Statistical Computing, Vienna (2021).

For manuscripts utilizing custom algorithms or software that are central to the research but not yet described in published literature, software must be made available to editors and reviewers. We strongly encourage code deposition in a community repository (e.g. GitHub). See the Nature Portfolio guidelines for submitting code & software for further information.

Data

Policy information about availability of data

All manuscripts must include a data availability statement. This statement should provide the following information, where applicable:

- Accession codes, unique identifiers, or web links for publicly available datasets
- A description of any restrictions on data availability
- For clinical datasets or third party data, please ensure that the statement adheres to our policy

All data are included in the paper and the Supplementary Material

Research involving human participants, their data, or biological material

Policy information about studies with human participants or human data. See also policy information about sex, gender (identity/presentation), and sexual orientation and race, ethnicity and racism.

Reporting on sex and gender	This information has not been collected
Reporting on race, ethnicity, or other socially relevant groupings	This information has not been collected
Population characteristics	This information has not been collected
Recruitment	This information has not been collected
Ethics oversight	This information has not been collected

Note that full information on the approval of the study protocol must also be provided in the manuscript.

Field-specific reporting

Please select the one below that is the best fit for your research. If you are not sure, read the appropriate sections before making your selection.

☐ Life sciences ☐ Behavioural & social sciences ☒ Ecological, evolutionary & environmental sciences

For a reference copy of the document with all sections, see nature.com/documents/nr-reporting-summary-flat.pdf

Ecological, evolutionary & environmental sciences study design

All studies must disclose on these points even when the disclosure is negative.

Study description	We assess the impacts of invasive parrot species on the structure and functioning of plant and bird recipient communities using a multilayer network framework. To do this, we quantify plant-bird interactions by monitoring tree and shrub species with fruits over a full annual cycle.
Research sample	Frugivorous bird species were included (families Columbidae: Columba palumbus, Corvidae: Coloeus monedula, Cyanopica cooki and Pica pica, Oriolidae: Oriolus oriolus, Sturnidae: Sturnus unicolor, Sylviidae: Sylvia atricapilla, Sborin, Curruca communis and C. melanocephala and Turdidae: Turdus merula and T. philomelos) and granivorous birds (families Passeridae: Passer domesticus, P. hispaniolensis and P. montanus and Fringillidae: Linaria cannabina, Carduelis carduelis, Chloris chloris, Fringilla coelebs and Serinus serinus) native to the study area, one feral species (Columba livia var. dom.), one natural invader (Streptopelia decaocto) and two invasive parrots, Psittacula krameri and Myiopsitta monachus.<br>Plant diversity in the study area was too vast to monitor comprehensively. We therefore conducted preliminary work to identify the main plant species utilized by birds for feeding. These selected species formed the basis for constructing the ecological networks and included native Mediterranean and exotic ornamental species with arboreal and shrub-like growth habits.
Sampling strategy	Over the course of a full annual cycle, we recorded bird-fruit interactions weekly. To maximise sampling while minimising pseudoreplication, each plant was GPS-recorded and observed for 30 minutes at each stage of its fruit phenology (unripe and ripe), beginning when foraging activity by birds was detected. During these sessions, foraging individuals were observed for 5 minutes, and all interactions with fruits were recorded. Monitoring occurred during the first three hours after sunrise and the last three hours before sunset, coinciding with the peak of bird foraging activity, while avoiding suboptimal weather conditions such as strong wind or rain. To maximize detection of interactions, we monitored as many individuals of each plant species as possible, covering the entire study area. To assess the adequacy of our sampling effort in capturing plant-animal interactions, we generated species accumulation curves for each plant species using the BiodiversityR 2.16.1 package
Data collection	Observations were made by 2-3 people using binoculars and telescopes, maintaining a minimum distance of 15-50m from the focal plant by Fernando Hiraldo, Jaume Izquierdo, Dailos Hernández-Brito (mainly) and Martina Carrete
Timing and spatial scale	Over the course of a full annual cycle (April 2022 and May 2023), we recorded bird-fruit interactions weekly.
Data exclusions	Interactions where birds only defleshed or wasted fruits were excluded from network analyses
Reproducibility	All attempts to repeat the experiment were successfull
Randomization	To maximise sampling while minimising pseudoreplication, each plant was GPS-recorded and observed for 30 minutes at each stage of its fruit phenology (unripe and ripe), beginning when foraging activity by birds was detected. To maximize detection of interactions, we monitored as many individuals of each plant species as possible, covering the entire study area.
Blinding	Blinding was not relevant for our study

Did the study involve field work? ☒ Yes ☐ No

Field work, collection and transport

Field conditions	Monitoring occurred during the first three hours after sunrise and the last three hours before sunset, coinciding with the peak of bird foraging activity, while avoiding suboptimal weather conditions such as strong wind or rain.
Location	Sevilla, Spain
Access & import/export	Not needed
Disturbance	No disturbance

Reporting for specific materials, systems and methods

We require information from authors about some types of materials, experimental systems and methods used in many studies. Here, indicate whether each material, system or method listed is relevant to your study. If you are not sure if a list item applies to your research, read the appropriate section before selecting a response.

Materials & experimental systems

n/a	Involved in the study
☒	☐ Antibodies
☒	☐ Eukaryotic cell lines
☒	☐ Palaeontology and archaeology
☐	☒ Animals and other organisms
☒	☐ Clinical data
☒	☐ Dual use research of concern
☐	☒ Plants

Methods

n/a	Involved in the study
☒	☐ ChIP-seq
☒	☐ Flow cytometry
☒	☐ MRI-based neuroimaging

Animals and other research organisms

Policy information about studies involving animals; ARRIVE guidelines recommended for reporting animal research, and Sex and Gender in Research

Laboratory animals	The study did not involve lab animals
Wild animals	Each plant was GPS-recorded and observed for 30 minutes at each stage of its fruit phenology (unripe and ripe), beginning when foraging activity by birds was detected. During these sessions, foraging individuals were observed for 5 minutes, and all interactions with fruits were recorded.
Reporting on sex	Birds were not sexed for the study
Field-collected samples	The study did not involve samples collected in the field
Ethics oversight	No ethical approval was needed as the study was observational.

Note that full information on the approval of the study protocol must also be provided in the manuscript.

Dual use research of concern

Policy information about dual use research of concern

Hazards

Could the accidental, deliberate or reckless misuse of agents or technologies generated in the work, or the application of information presented in the manuscript, pose a threat to:

No	Yes	
☒	☐	Public health
☒	☐	National security
☒	☐	Crops and/or livestock
☒	☐	Ecosystems
☒	☐	Any other significant area

Experiments of concern

Does the work involve any of these experiments of concern:

No	Yes	
☒	☐	Demonstrate how to render a vaccine ineffective
☒	☐	Confer resistance to therapeutically useful antibiotics or antiviral agents
☒	☐	Enhance the virulence of a pathogen or render a nonpathogen virulent
☒	☐	Increase transmissibility of a pathogen
☒	☐	Alter the host range of a pathogen
☒	☐	Enable evasion of diagnostic/detection modalities
☒	☐	Enable the weaponization of a biological agent or toxin
☒	☐	Any other potentially harmful combination of experiments and agents

Plants

Seed stocks	Not applicable
Novel plant genotypes	Not applicable
Authentication	Not applicable
